# Reducing Health Effects on Deceit in Heterosexual Sexual-Affective Relationships: The Impact of the Preventive Socialization Program (PSP)

**DOI:** 10.3390/ijerph19042274

**Published:** 2022-02-17

**Authors:** Lidia Puigvert-Mallart, Roger Campdepadrós Cullell, Josep Maria Canal, Carme García-Yeste

**Affiliations:** 1 Department of Sociology, University of Barcelona, 08034 Barcelona, Spain; lidia.puigvert@ub.edu; 2 Department of Business, University of Girona, 17003 Girona, Spain; roger.campdepadros@udg.edu; 3 Area of Educational Sciences, Valencian International University, 46002 Valencia, Spain; juscanal@gmail.com; 4 Department of Pedagogy, Rovira i Virgili University, 43007 Tarragona, Spain

**Keywords:** deceit, sexual-affective relationships, masculinities, health, preventive socialization program

## Abstract

Background: Health research has provided robust evidence of the negative effects caused by facing deceit in sexual-affective relationships. In this regard, several analyses have been conducted addressing psychological, family therapies, and educational interventions to improve marital problems. On the other hand, many investigations have addressed how the preventive socialization program (PSP), framed on the analysis of a dominant coercive discourse that is connected with the promotion of specific traditional masculine models, impacts on young people’s relationships free of violence. However, the link between deceit, health, and the PSP has not yet been analyzed. To cover this gap, a qualitative study has been performed with a methodology framed on the communicative approach. We enrolled heterosexual women and men aged 25 to 42 years old who have been in contact with the PSP and have experienced or know of any cases of deceit. Two different analytical categories emerged from the analysis, which distinguishes between exclusionary and transformative dimensions. Findings show that the majority of people involved in PSP were able to understand the reasons for deceit as well as to take decisions in their further sexual-affective relationships. Therefore, this study corroborates that access to scientific evidence in the framework of PSP is positively impacting interviewees’ health status.

## 1. Introduction

Deceit in sexual-affective relationships is a topic that is globally investigated in psychology and health, mainly demonstrating that is a phenomenon that can cause several health problems. Thus, research shows how deceit in these kinds of relationships could cause mental disorders, such as stress, anxiety, and depression [1,2,3]. Other analyses corroborate how deceit increases the risk of having HIV, and it is particularly relevant data in young males [4,5]. Finally, the relation between deceit and suicide has been studies [6]. 

Drawing on this evidence, the present study aimed at going beyond to conduct a descriptive analysis of the effects of deceit, analyzing how the preventive socialization program (PSP, hereinafter), focused on the study of the effects of a coercive discourse and the promotion of dominant traditional masculinities, is contributing to combat the abovementioned health effects [7,8,9]. PSP is a research line based on the knowledge accumulated through two decades of competitive investigation aimed at discovering reasons for the prevalence of gender-based violence in society. Among the findings is the existence of a coercive dominant discourse that links attraction with violence in dominant traditional masculinities [10], and how this link is affecting the socialization process of heterosexual men and women in their sexual-affective relationships. The shaping of this kind of socialization process is directly becoming a risk factor for gender-based violence victimization. PSP has other relevant components, which include scientific knowledge aimed at contributing to creating alternative socialization processes, such as the shaping of new alternative masculinities [11]; the understanding of the mirage of upward mobility [12]; the role of friendship and solidarity to prevent violence [13]; the dialogic reconstruction of memories linked to affective and sexual relationships [14]; and the definition of a language of desire directly connected to kindness and attractiveness [15]. 

In the present article, we will detail the incidence of the PSP, but pay attention to how it is contributing to a deeper understanding of deceit in sexual-affective relationships and the role of masculinities in that process. In parallel, we will contrast the steps taken by PSP and the existing literature that analyzes different interventions from psychology and education that are tackling deceit. 

### 1.1. State of the Art

A literature review on forms of preventing deceit and improving subjects’ wellbeing has identified the existence of several analyses of interventions which are focused on: psychological and families therapies addressing how to approach infidelity in couples [16,17,18] and educational interventions to improve marital problems [19,20]. 

#### 1.1.1. Psychological and Family Therapies 

The investigation on psychological therapies suggests the existence of different approaches. Firstly, there is a study conducted by North, Shadi, & Hertlein [17] who explored the effect of a specific therapy, the so-called Trust and Deception Focused Genogram, which is mostly used in family therapies aimed at working patterns of behavior and communication to approach deceit within family members. The therapies based on Genograms pay attention to patterns of behavior and heterosexual couple’s communication aimed at exploring the reasons for deceit as well as identifying possible solutions. The therapist started to implement the aforementioned therapy to help the couple to identify the effects that this deception had on both. After describing these aspects, the article presents some insights into the social impact achieved by this therapy. This case was characterized by infidelity carried out by the husband which generated a climate of mistrust in the relationship. Drawing on this situation, the therapist started to implement the aforementioned therapy to help the couple to identify the effects that this deception had on both. After completing the Genogram, significant progress was reached. On one hand, the couple could identify what was the meaning of trust for both sides, particularly the husband could learn the impact that deception caused on his wife was. The authors of the study concluded that the implementation of this therapy was useful for better managing the relationship and for fostering a more appropriate form of communicating affection. In particular, paying attention to the changes in men’s way to interact and express their feelings with their wives. 

Within these studies which explore the preventive effects of psychological therapies, there is one that follows an integrative approach that considers the examination of people’s traumatic response and interpersonal forgiveness [16]. The analysis of the implementation of this therapy illustrates two different kinds of impacts. Firstly, participants involved in the therapy show a modest improvement regarding reducing levels of anxiety and depression. Secondly, the therapy helps the couple to evaluate its situation and consequently prevent possible further problems in this regard. This therapy includes three different stages which are designed to deepen the understanding of causes of deception and the way to go beyond it. Thus, in the first stage, the initial impact caused by deception is approached to learn to manage emotional and decision-making skills. The second stage is based on the analysis of factors within the couple which have increased vulnerability to having an affair. Finally, in the last stage, the therapy is focused on exploring personal beliefs about forgiveness and connecting these to recovery from infidelity. 

Another research that explores the impact of a similar therapy is conducted by Negash, Carlson, and Linder [21]. They evaluated the effects of the combination of two psychological therapies: Emotionally focused therapy (EFT) and Eye movement desensitization and reprocessing (EMDR), which were employed to treat the trauma suffered by couples who have experienced infidelity. This intervention implies eight phases that include different strategies, such as gathering client history, desensitization which means that patient is asked to allow their mind to “float”, body scan, among others. The examination shows interesting results concerning identifying conflicts that have never emerged before and started to solve problems between them. 

#### 1.1.2. Educational Interventions

As we have mentioned before, there is another kind of investigation which deepens the effects of specific educational interventions addressed to face deceit [19]. Among these studies, there is one that looks at the impact of a three-credit university course implemented in 380 colleges university students of the United States. These students were invited to participate in the study and were selected because they assured to be in an exclusive heterosexual romantic relationship. After the course was developed, students who were interviewed said that they have reduced the frequency of extradyadic involvement, understood as a physical or emotional relationship that occurs outside of an existing romantic relationship [22]. However, the results also suggest that this kind of intervention has more effect on men than on women. Thus, at the end of the intervention, women show high percentages of extradyadic involvement than men.

There is research that also studied in detail an educational intervention; in this case, it is referred to a marriage education action which is addressed to married heterosexual army couples in the United States. This intervention was centered on the realization of a workshop divided into two parts: one-day training and a weekend retreat at a hotel of 14 hours of training [20]. Both training interventions were based on psychological research which identified factors that drive a healthy marriage [23]. The study was framed on a sample composed of 662 couples who were randomly assigned to this intervention following a previous and post-analysis and also an evaluation after one year of its implementation. A total of 23.4% of the couples selected for the sample reported a history of infidelity during their marriage. Findings showed that couples with a history of infidelity have problems with communication before the intervention; nevertheless, when the intervention was executed, a relevant improvement in their marital satisfaction and communication skills was identified. 

#### 1.1.3. Prevetive Socialization Program 

PSP has a different objective than the abovementioned therapies because it does not want to change people’s choices and relations; its purpose is to bring the scientific evidence closer to subjects aimed at approaching a better understanding of deceit in sexual and affective relationships. Thus, PSP pays attention to the role of social interactions and socialization as elements that influence these kinds of decisions. On the other hand, the PSP coincides with the educational interventions on the relevance given to knowledge that helps to understand the reason for deceit in affective relationships. However, as it will be deeply developed along with the article, PSP pays attention to this knowledge, which connects the creation of a coercive dominant discourse and the shaping of a traditional model of masculinity as key reasons to acknowledge deceit [7,8,9]. In fact, this element, directly connected with the already introduced socialization processes and interactions, is less threatened in the analyzed educational interventions. Drawing on these key differences and considering the previous investigation on this field, this study will be oriented to answer two objectives: to detect the health effects of deceit; and to identify how the PSP contributed to a better understanding of deceit and consequently to be more aware of its effects on subjects’ health status. As it will be detailed in the results section, knowledge of the scientific evidence of PSP drives interviewees to take better decisions related to their health.

## 2. Materials and Methods 

The methodology employed in this study follows a communicative approach which is characterized by the establishment of an egalitarian dialogue between the interviewees and the interviewers [24,25]. Communicative Methodology (CM, hereinafter) starts on the premise that any subject has a capacity for action and communication, and with their expertise from daily life or their professional background can contribute to creating intersubjective knowledge. Through this dialogue, interviewers provide scientific knowledge and interviewees contribute with their lifeworld, which is connected on their common sense. Another characteristic of CM is its purpose of obtaining knowledge addressed to social transformation. This means that data analysis is aimed to detect these transformative dimensions which are contributing to facing barriers and getting social impact [26]. 

In the present study, both characteristics have been articulated; therefore, specific explanations about research findings on masculinities and deceit have been presented to interviewees. Then, thanks to this exchange of knowledge, subjects involved in the research could contrast findings from literature with their own experience with the PSP. In communicative methodology, when this kind of exchange is developed, it contributes to creating knowledge that provides transformative elements to combat discrimination and inequalities. For instance, previous research that employed the CM and studied gender-based violence in Spanish universities created a knowledge that was scaled up to university policies [27,28,29].

### 2.1. Data Collection Method and Sample

This has been a qualitative study based on the development of 10 communicative daily life stories carried out from August to October 2019. The 10 subjects have all heterosexual preferences and they were born in Spain. They include six women and four men whose age range was from 25 to 42 years old. These participants have been recruited because they have been in touch with the scientific evidence of the PSP at the least in the past three years. In addition, they have been contacted through the research team whose expertise with the PSP facilitated access to the interviewees. All of them have high-education backgrounds, mostly have a Ph.D., Master’s degree, or Bachelor’s, and belong to medium socio-economic status. This similarity on the profiles is directly related to the connection with the PSP, which is a research approach that is mainly performed in higher education and teacher training contexts. 

All of them have read the evidence on preventive socialization and have been involved in investigations in this field. For this reason, they have been selected for the study as well as for having experienced, or know close people who have experienced, deceit in their sexual and affective relationships. One of the biases which we considered during the data collection process was the homogeneity of the profiles selected, but we prioritized that subjects have followed this training process on the evidence collected from PSP. More details on the subjects selected are summarized in Table 1, describing their profile and their connection with deceit and involvement in the PSP. 

### 2.2. Data Analysis

The 10 communicative life stories were transcribed verbatim, including all the dialogues and reflections between researchers and interviewees. Spanish and Catalan were the languages employed in those life stories, so then the research team translated the selected quotes into English. This translation was conducted after the data analysis respecting the original meaning and following the English words employed in previous research where the PSP was studied. Thus, the data analysis was focused on the narratives that subjects realized about the health effects of deceit and the impact of knowing the evidence of the PSP. Starting from this information, quotes were selected and hand-coded from each life story aimed at responding to the purposes of the investigation. This codification process was inductively realized considering the analytical premises of the communicative methodology and the theories linked to PSP and deceit. Therefore, the quotes were differentiated between two main codes: exclusionary and transformative dimensions, which is the analytical strategy followed in the CM. The exclusionary dimensions were defined by CM as the barriers that hinder social change, so in our research are interconnected with theories that approach the health effects of deceit. On the other hand, CM defines transformative dimensions as those actions, realities, and practices that contribute to overcoming any barrier. In the present study, these dimensions are related to the impact of the PSP. Particularly, which makes subjects deeply understand the reasons and influence of deceit connected with the shaping of dominant coercive discourse and the reproduction of traditional dominant models of masculinity. As it will be more detailed in the results section, these transformative dimensions are split into four different subdimensions: normalizing narratives of deceit, ethical people are perceived as not attractive, being aware of their own health status, and making sense of the past. 

### 2.3. Ethical Procedures

The present study has considered the ethical guidelines established by the European Commission in the implementation of responsible research [30]. In this regard, we have informed interviewees about the objectives of our investigation and a consent form has been provided and was signed by all participants. In addition, to guarantee subjects’ identity, their names have been anonymized and we have changed them by pseudonyms. Additionally, the study was evaluated by the Ethical Committee of CREA—Community on Excellence for All—which validates the methodological instruments employed and the data analysis conducted. 

## 3. Results

The findings obtained through the analysis of the communicative life stories identify two main contributions which were previously introduced in the methodological section: health effects of deceit (exclusionary dimension) and understanding in depth the influence of deceit and the access to scientific evidence related to PSP (transformative dimensions).

### 3.1. Negative Health Effects of Deceit

As has already been demonstrated, being deceived has negative effects on physical and mental health [1,2,3]. In the present study, this reality is also evidenced in Ines’story. She explained the consequences on her health status of having faced deceit. She explained the effects of starting a relationship with a man who permanently undervalued her and showed traits of dominant traditional masculinities: 

I had a lot of stress, not only psychologically but physically, a lot. In fact, I developed a problem of hyperthyroidism, in the time I was in this relationship. I am convinced that it was directly connected with the level of stress I had when I was in this relationship marked by deceit. 

Bogdan, also reports the impacts of having lived through deceit in a long relationship. These impacts were directly related to his self-esteem that influenced his eating habits. He lost motivation for doing anything because he felt that he was not deciding about his life: 

I didn’t trust her at all, and there was always a lot of tension. After I ended the relationship with that person, I remember that I also felt guilty for being in a relationship like that but I kept going. (…) Self-esteem went to shit, I had 0, but I had no control over anything. I let myself go, and I have 0 confidence. I took much less care of myself, in terms of eating habits, fatal, I didn’t control anything and I also had a self-destructive tendency that I didn’t care about, I didn’t see the point of anything. I was not motivated at all.

Similarly, Blai’s explanation of the consequences of being deceived and having deceived shows other health-related problems. He argued that the deceit created a no-sense scenario in his life which drove him to follow toxic masculinity models that were very connected to abuse of alcohol: ‘A crisis of meaning which drove me to drift, in terms of health. Well, l drank more alcohol because I needed to drink more’. 

### 3.2. Understanding in-Depth the Influence of Deceit

One of the transformative elements which emerge in the interviews related to the understanding of the influence of deceit is the identification of the coercive dominant discourse. As it was identified by previous studies, the existence of this discourse pushes people to be socialized in masculinity models that link attractiveness and desire with violence. In fact, this is one of the main issues tackled in the PSP, as has previously been introduced [8,31]. The analysis of some of their life stories reveals that knowing the existence of this coercive dominant discourse contributed to identifying the influence of deceit in their behavior and health well-being. Among this identification of the coercive dominant discourse, two main aspects are stressed: the normalization of narratives that justify deceit as something beneficial and how ethical people, who no deceit, are perceived as non-attractive. 

#### 3.2.1. Normalizing Narratives of Deceit

Eulalia’s words in her story described how the coercive dominant discourse was common in her context. She explained that she planned to travel to Guatemala for International Cooperation and how normalized talking positively about deceit was among her colleagues and parents. After knowing the evidence of the PSP, she is more aware of the silence that her friends had when her boyfriends was deceiving her: ‘There was an environment that normalized this, they assumed that if I had gone to Guatemala, I had to make out with someone from there. And anyone positioned that my new couple was cheating me constantly’. 

Patricia’s story has several similarities with Eulalia’s one. She narrated that the coercive dominant discourse influenced her first sexual-affective relationship. Nowadays, as she recognized, knowing the evidence of PSP contributed to a better understanding of why her context normalized deceit and branded her as a conservative person: 

The idea is that he was with other people but in a context that was deception, but the problem that was there was a very ‘postmodern’ context, any attempt to say that something was not right. They said that you are very conservative …You could not even say they’re cheating me. Then any attempt to say that this situation was horrible was impossible, they said that I was conservative. 

#### 3.2.2. Ethical People Are Perceived as Not Attractive

In the line of identifying the effects of coercive dominant discourse, interviewees explained how being in touch with the PSP has also contributed to comprehending why becoming an ethical person, in terms of not deceiving, is perceived as non-attractive. For instance, in Igone’s story, a reflection about that reality is illustrated, particularly referring to the excitement for deceiving, for feeling proud for deceiving, and for having ethical principles which are contrarily perceived as something boring. Igone illustrates with her experience the feelings she had when deceit happened around her: 

He felt very proud, he was very proud. He explained it with a lot of excitement. Apart from that, deception was perceived as very attractive, then it was completely justified. She and all, but he is the one with the girlfriend: ‘It is not my fault it is your responsibility; I do what I want. In any case, it will be he who will see what he must do’ (…) Normalization and if people push yourself to deceive—‘it does not matter; I have fun with him’. If you liked a boy who had a girlfriend and if you showed ethical principles, it didn’t matter. 

In Pere’s story, the influence of male competitiveness in his understanding of deceit appears. In that case, he identifies the effects of this kind of competitiveness in becoming more successful and a better man if he is deceiving: ‘It was established as a competition (…) because of course, you demonstrated that you were better than the other, it is the excitement for deceiving someone who already has a partner’.

### 3.3. Access to Scientific Evidence

As it has been already introduced in the methodology, the access to scientific evidence interconnected with the PSP has reported significant knowledge to the interviewed subjects, which drives them to be aware of their health status and deepen from another perspective to their past relationships. Thus, two different categories have been shaped in this section: how knowing the evidence of the PSP contributes to being aware of their health status and that the past makes sense when evidence approached in the PSP is internalized. 

#### 3.3.1. Being Aware of the Health Status 

As abovementioned, interviewees pointed out that having access to the evidence of the PSP became important to understand more why deceit happened and the effects in terms of health. In addition, it also helps them to decide with more knowledge about their further relationships, reinforcing their identity and empowering themselves. For instance, Eulalia insisted on the relevance to talk with the evidence of the PSP as a tool to understand what she was living through in her relationships. She felt coerced by a discourse that drove her to deceive or to accept being deceived. Thanks to this knowledge, she is currently noticing a comforting feeling linked to having positive values and principles: 

This saved my life (…) Then, talking about that, and understanding it, above all, understanding all the coercion patterns. For example, the condoms box was something I had overlooked and remembering things, oh my god, is true. (…) Thanks to speaking a lot and reflecting on it very much, the issue of attraction to violence, yes, I remembered a lot of details. And this process helped me a lot, to disassemble this story, both the history of Guatemala, and the relationship that I had afterward. Yes, I think that at some point in my life I had the principles, the good values inside, but I felt that it had been ripped apart from me. And now I feel like I have them again, this desire for good things. I feel radically different. 

Blai’s narrative demonstrates changes in physical and psychological well-being. He described his experiences in the past where deceit was normalized and how he connects that period with following specific habits, such as high levels of alcohol consumption. At present, he said he is more aware of these practices and he feels more self-confident about his decision to not drink: 

It was an agony of ups and downs because on the one hand I saw the rejection that I had and this caused me permanent unhappiness. (…) A crisis of meaning drove me to feel lost in terms of health well-being. To drink more alcohol, because in the past I needed to drink more when I got out because I thought that socializing was easier. And now, I get out and I don’t need to drink, and I see the ridicule that I did at that time. Then, at the psychological level, I have improved a lot, because I feel more self-confident. 

Lastly, it is important to highlight Xavier’s steps after having access to the PSP. He experienced episodes of anxiety because he did not understand why his male friends were always pushing him to deceive. He understood the nature of this masculine pressure when he started to be in contact with the scientific evidence. In addition, having access to this knowledge empowered him to be braver when any situation like that happens: 

If I think how I was a few years ago before I could access all this evidence, I felt really very lost at many levels and that situation drove me to great anxiety, and I was very unhappy. I had very destructive thoughts. At the moment I can talk about that and analyze it from scientific evidence (…) I started to know that behind this there is research and a foundation, and also that everything has a meaning, that when you talk about it everything fits. It helps you to overcome any difficulty. It gives you a point of support to have a courageous attitude in front of certain things.

#### 3.3.2. Making Sense of the Past 

Through dialogues with her colleagues, based on the knowledge acquired in the PSP, Patricia understood what happened in her past relationships. Now, she has understood the reasons of those situations, which has helped her to be more self-confident and to know how to proceed when any situation can affect her psychological well-being. This new analysis about the men she chose, empowers her in the present: 

In the past, I was a very insecure person concerning appeal (…) And now, thanks to PSP, I do not have these insecurities, I am far from that feeling. I have great self-esteem, assuming how I am. It is also true that when I start with a story that can affect me psychologically, immediately I speak about it. 

Similarly, Bogdan expresses their thoughts about past relationships and shares with other interviewees a new comprehension of those relationships. Likewise, Bogdan confesses that he underestimated himself in his previous relationship with a girl. Later, after a few years, and his contact with the PSP, he understood situations where he was not asserting himself and how this did not awake attraction in his girlfriend: 

From the start I gave everything and put her on a pedestal and thats it, I’ll be there for the rest of my life and I’ll do whatever you ask me to do, and I was putting myself very low. And I was aware that these things were not attractive at all. Of course, I understood this later, knowing the evidence. 

Finally, Blai’s story also shows this dynamic of realizing past moments linked to deceit. He explains how sharing with other people the evidence of the PSP contributed to analyzing differently a past relationship where deceit was present. Blai affirms that when he experienced that situation he did not understand what was happening, but he is currently finding out thanks to the PSP: 

I remember for example one day, concerning cheating, I was making out with a girl, and at a certain moment she told me to stop and suddenly everything stopped and she was very upset and she couldn’t explain what was happening to her. At the time I thought it was a health issue, that she couldn’t go on, but the reality is that she was cheating on her boyfriend. And this has been talking to people who also had this evidence.

## 4. Conclusions

The study conducted here is complementing previous analyses from two different research approaches. The first one is related to the consequences of deceit in affective and sexual relationships [1,2,3]. The second one concerns the effects of implementing specific interventions addressing deceit, mainly framed on psychological and educational perspectives, in people’s well-being [4,16,17,32]. Thus, our results corroborate how health problems appear when sexual and affective relationships are marked by deceit, but they also illustrate the relevance of accessing the scientific evidence of the PSP to have more knowledge for taking decisions that connect affective and sexual relationships with health. 

Concerning the perspective of interventions, there are some links with our study which should be underlined. For example, Baquero, Santos, and Ocampo’s [18] study confirmed how the implementation of a psychological intervention, based on Baucom et al.’s [32] stages of treatment, generates individual changes connected with patients’ needs and social relationships. Quite connected with this finding is the one obtained by our research that demonstrates how being in touch with the PSP contributes to a widening of heterosexual men and women’s knowledge about desire and attraction in sexual-affective relationships. In other words, the PSP gives responses to understand differently which situations and interactions can help—or not—to take decisions and choices that would be favorable for these relationships. Hence, this result opens new opportunities for the inclusion of desire and attraction in psychological interventions.

Another effect is evidenced in the investigation conducted by Negash, Carlson, and Linder (2018), that pays attention to psychological therapy which helps patients to identify conflicts in their relationships that have not been tackled before [21]. This identification was very useful for them to start a conflict resolution process that improves their relationship. In this line, in our study, it has been stated how the PSP is also contributing to identifying new elements which make a different understanding of past relationships. Particularly, the PSP helps to recognize the role of coercive dominant discourse and the traditional models of masculinity in the social reproduction of relationships framed on deceit. 

Regarding the amount of research addressing educational interventions, the investigation performed by Braithwaite et al. [19] shows how a training course addressed to university students contributes to reducing extradyadic involvement. In that case, the influence of training is an issue that also emerges in our study; however, in the PSP, the effects on improving well-being and changing past habits are demonstrated, which is an aspect not mentioned in the previous studies on this field. Therefore, PSP gives new elements to take into account in further designs of training courses with health educators. 

## 5. Discussion

Despite all the above mentioned connections, complementary aspects arise from our analysis on the effects of being trained in the PSP. Firstly, the PSP enables a new interpretation of sexual-affective relationships which contributes to a new understanding of them and the incidence of deceit in health well-being. This new interpretation generates critical thinking that provides renewed capacities to choose non-toxic relationships and alternative models of masculinity. The latter means that men empower themselves to not reproduce hegemonic and traditional masculinities, and at the same time encourage heterosexual women to choose alternative models of masculinity. Nevertheless, a separate analysis between the effects of PSP on men and women for further research would be relevant to deepen the different impacts reached by the research approach.

Secondly, PSP is an ongoing research line because it is permanently updated through new investigations in the field. Concerning this last point, this updating process is enriched through an intersubjective dialogue that is established when evidence on the PSP is shared with other people involved in the field. Following Gómez’s words [33], a precursor researcher of PSP, there is a possibility to construct sexual-affective relationships which generate illusion and motivation, but it is necessary scientific knowledge and personal commitment.

Lastly, it is important to underline that our study presents some limitations, one of these is connected with the homogeneity of the sample which prevents us to make wide inferences about the steps achieved by the PSP. Therefore, the results cannot be generalized and extrapolated to all people who have been in touch with the PSP. Another limitation is related to the continuous updating of the scientific evidence of the PSP, which makes it difficult to have comprehensive knowledge by all the subjects involved in the sample.

## Figures and Tables

**Table 1:** Description of the profiles of the subjects involved in the study.

Pseudonym	Profile
Maria	She was deceived being a teenager and this situation drove her to unhealthy relationships. She started a Master in Sociology where she knew the PSP.
Yolanda	She was in a relationship for 5 years until she was deceived. Thanks to being involved in a feminist research group she knew the PSP, and this knowledge helped her to understand the reasons for her infidelity.
Xavier	In his twenties, he had a friendship where deception was normalized, and he decided to be involved in an egalitarian men’s movement. In this movement, he knew the PSP.
Blai	He had different relationships framed on deceit. After that, he started a long relationship with a feminist woman who encourages him to learn more about the PSP. Currently, he is involved in an egalitarian men’s movement.
Bogdan	He was deceived in a long relationship, and this influenced his self-esteem. He had several mental disorders. He started a master’s degree where he knew the PSP.
Eulalia	She deceived and was deceived. She was a victim of sexual harassment in the university, and she started to be in contact with the PSP in a master’s degree and then in her Doctorate. She presented a dissertation focused on the social impact of Social Sciences and Humanities.
Pere	He deceived and was deceived. He was in a long sexual-affective relationship where deceit was normalized. In the last 7 years, he started to be involved in an egalitarian men’s movement where he discovered, through scientific discussions, the basis of the PSP.
Patricia	She deceived and was deceived. She was involved in different toxic relationships marked by deceit and violence. She is currently a university professor in the field of education and gender studies. One of her research interests is the effects of the PSP in higher education.
Igone	She did not deceive, but her past friends had relationships based on deceit. She was involved in a kind of friendship where deceit was promoted and normalized. Recently, she started a master’s degree and a Ph.D. where the PSP is one of her main research focus.
Ines	She has been deceived in two of her previous sexual-affective relationships. She is currently involved in a feminist women’s group. She is a university professor, and she is studying the effects of interventions based on the PSP in psychological well-being.

## Data Availability

The data presented in this study are available on request from the corresponding author. The data are not publicly available due to ethical restrictions.
